# Getting fat or getting help? How female mammals cope with energetic constraints on reproduction

**DOI:** 10.1186/s12983-017-0214-0

**Published:** 2017-06-12

**Authors:** Sandra A. Heldstab, Carel P. van Schaik, Karin Isler

**Affiliations:** Department of Anthropology, University of Zurich, Winterthurerstrasse 190, 8057 Zurich, Switzerland

**Keywords:** Allomaternal care, Cooperative breeding, Body fat, Paternal care, Helping behaviours, Reproduction, Allonursing, Provisioning

## Abstract

**Background:**

Fat deposits enable a female mammal to bear the energy costs of offspring production and thus greatly influence her reproductive success. However, increasing locomotor costs and reduced agility counterbalance the fitness benefits of storing body fat. In species where costs of reproduction are distributed over other individuals such as fathers or non-breeding group members, reproductive females might therefore benefit from storing less energy in the form of body fat.

**Results:**

Using a phylogenetic comparative approach on a sample of 87 mammalian species, and controlling for possible confounding variables, we found that reproductive females of species with allomaternal care exhibit reduced annual variation in body mass (estimated as CV body mass), which is a good proxy for the tendency to store body fat. Differential analyses of care behaviours such as allonursing or provisioning corroborated an energetic interpretation of this finding. The presumably most energy-intensive form of allomaternal care, provisioning of the young, had the strongest effect on CV body mass. In contrast, allonursing, which involves no additional influx of energy but distributes maternal help across different mothers, was not correlated with CV body mass.

**Conclusions:**

Our results suggest that reproducing females in species with allomaternal care can afford to reduce reliance on fat reserves because of the helpers’ energetic contribution towards offspring rearing.

**Electronic supplementary material:**

The online version of this article (doi:10.1186/s12983-017-0214-0) contains supplementary material, which is available to authorized users.

## Background

Reproduction is energetically very expensive [1, 2] and several studies show that the amount of food available and hence the total amount of energy invested by the mother influences reproductive success in female mammals. Provisioning by humans generally leads to higher reproductive rates, shorter lactation periods, and shorter inter-birth intervals [3–5]. In natural animal populations, higher food abundance leads to higher birth rates [6–11]. In contrast, food restriction may delay sexual maturation and among adults may inhibit mating behaviour [12–14] or even produce acyclicity or anoestrus [15, 16].

In mammals that evolved in seasonal environments and thus face periods of food scarcity, a female’s ability to bear the energy costs of pregnancy and lactation, and thus her reproductive success, may be affected by the amount of body fat she can deposit. That stored body fat plays an essential role in female reproduction has been proposed previously within the capital-income-continuum concept (for a review see [17]) and empirical evidence for this idea is abundant. For instance, in rhesus macaques (*Macaca mulatta*) and moose (*Alces alces*), the size of maternal fat stores positively affects pregnancy and birth rates [18, 19]. Furthermore, numerous studies show that heavier and fatter mothers produce heavier offspring that grow faster and are more likely to survive, suggesting that females in better body condition are able to allocate more stored resources to reproduction [20–25]. Finally, several studies in seals show that body fat is essential for lactation as seal mothers lose more than 50% of their stored body fat until the end of lactation ([26] and references therein). Significant seasonal fattening in females may also be found if they do not reproduce, e.g. to buffer environmental food fluctuations [27, 28]. However, because reproductive seasons and experienced seasonality in food intake are generally interrelated, it is usually impossible to disentangle these two reasons for body fat storage [29–33]. Female polar bears (*Ursus maritimus*) offer an extreme example of this. They store body fat to hibernate due to adverse environmental conditions for up to 8 months while simultaneously meeting the nutritional demands of gestation and lactation during this fasting period [23].

But the positive effect of fat stores on fitness is counterbalanced by their costs. Large fat reserves increase the energy costs of locomotion due to higher body weight [34–37], and also reduce agility and speed and so may compromise fitness by increasing predation risk or decreasing hunting success [38–42]. Furthermore, in arboreal species, body fat may also impede terminal branch feeding [43]. Indeed, arboreal species are less prone to store fat than terrestrial ones [44]. Therefore, we hypothesize that female mammals should minimize the amount of fat stores if they have an alternative to fuel their reproductive success.

All other things being equal, the energetic burden of reproduction on reproductive females is reduced when the costs of reproduction are distributed over several individuals. Thus, in species where other individuals provide energetic costly allomaternal care behaviours, breeding females might need to store less energy in the form of body fat themselves and could avoid the locomotion and predation costs resulting from high amounts of body fat. Allomaternal inputs are found in many mammals, comprising behaviours such as provisioning, carrying, huddling or communal nesting, babysitting, and protection from predators or defence of resources against conspecifics. The effects of such allomaternal care on offspring survival or fertility have been demonstrated within and between species [45–51]. One likely mechanism underlying this effect is load-lightening of pregnant or lactating females by helpers (‘load-lightening’ hypothesis [52]) which has been demonstrated in meerkats [53], callitrichids [54, 55] and siamangs [56]. This load-lightening effect has also been demonstrated in some species with facultative helping, where females can rear their pups solitarily, but under certain conditions share care for the young with one or more additional individuals. For instance, female prairie voles (*Microtus ochrogaster*) and pine voles (*Microtus pinetorum*) had shorter interlitter intervals in family groups consisting of the breeding pair and former offspring compared to families without previous offspring [57, 58]. In striped mice (*Rhabdomys pumilio*) living in the succulent karoo, offspring grew faster when the father was present, which may indirectly benefit females when young are weaned earlier [59]. In females of a facultatively cooperative breeding bird species, the splendid fairy-wren (*Malurus splendens*), the presence of helpers has been shown to increase survival of the breeding females and reduce the time for these females to renest after a brood [60]. Lastly, in another facultative cooperative breeder, the western bluebird (*Sialia mexicana*), the presence of helpers allowed the breeding female to lower her feeding rate, while nestlings still received more feeds at nests with helpers compared to nests without helpers present [60]. In sum, there is ample empirical evidence that distributing the costs of reproduction over two or more individuals yields an energetic benefit for mothers or offspring. We do not distinguish between the two, as a net fitness effect can be obtained by either.

Allonursing, the nursing of non-filial offspring, is another form of care that has been observed in every major mammalian lineage [61, 62]. However, allonursing events within a species are generally rare. For instance, in tufted capuchin monkeys (*Sapajus nigritus*) allosuckling accounted for 13% of all suckling events [63], in South American fur seals (*Arctocephalus australis*) for around 3% [64], and in red deer calves (*Cervus elaphus*) allosucking was even less common [65]. Furthermore, the rejection rates of suckling of non-filial offspring are high. In guanacos (*Lama guanicoe*), for example, the rejection rate to non-filial offspring nursing attempts was three times higher than the rejection rate to filial nursing attempts [66]. Although allonursing may confer social benefits to the allonursed young [63, 67], the energetic benefits for offspring or mother are unclear. First, allonursing is more likely to occur when several females breed concurrently [62] and hence all females simultaneously bear the costs of reproduction. Therefore, the idea that allonursing functions as load-lightening mechanism for lactating females cannot apply [68], and instead allonursing may serve to more evenly divide maternal energy investment across different mothers [69]. Second, several studies show no apparent energetic benefits of allonursing for recipient offspring and/or mothers. For instance, red deer calves sucking only from maternal hinds increased faster in body weight than calves sucking maternal and non-maternal hinds [65]. Another study found no evidence that allonursing provides benefits to meerkat pups (*Suricata suricatta*) or mothers [70]: pups that received allonursing were not heavier at emergence and did not have a higher survival rate than pups that did not receive allonursing. Mothers whose litters were allonursed were not in better physical condition, did not reconceive faster and did not reduce their own nursing investment compared to mothers who nursed their litters alone. To sum up, allonursing does not necessarily provide energetic benefits for the mother or offspring.

With the exception of allonursing, all other allomaternal care behaviours can be performed by all sorts of helpers in cooperatively breeding species, including fathers or non-breeding group members. Whereas the help provided by adult males (potential fathers) might be unaffected by their body condition [71] or food abundance [72], other non-breeding group members generally adjust their helping efforts in relation to their body condition. Furthermore, subordinates can also start to breed themselves, in which case their help to the dominant female could end abruptly or be minimal to begin with [73, 74]. These results suggest that paternal care is more reliable and thus more important for females than the help of others. On the other hand, in cooperative breeders more helpers than just the father might be around to take over the energetic costs of female reproduction. The optimum amount of body fat stored by a female may therefore vary depending on whether they receive no care, paternal care or additional help from several non-breeding group members.

The aim of this study is to test whether energetic contributions towards offspring rearing through costly care allow reproductive females to reduce the amount of energy (stored as body fat) they themselves need to invest. As a proxy for the seasonal tendency to store body fat, we use data on seasonal body mass variation within a year, the coefficient of variation (CV) in body mass, which has been shown to correlate with the amount of body fat within [44] and across species (PGLS: *P* = 0.03, *N* = 8, λ = 0, R^2^ = 0.56, β = 0.19, S.E. = 0.07, *t* = 2.74, calculated from data in [44]). Compared to single body fat values obtained from cadavers, CV body mass captures seasonal fluctuations, allows for a larger sample size for each species and can also be collected for wild animals [75]. In total, both reliable information on the nature and extent of allomaternal help and sufficient data on annual variation in body mass was available for 87 species from 9 mammalian orders.

We expect that an increased energetic contribution in the form of allomaternal care provided by the male or non-breeding group members is negatively correlated with annual variation in body mass in females, because storing fat and allomaternal subsidies independently stabilize the energetic costs for female reproduction. To test this prediction, we explore the effect of different types of allomaternal help on annual body mass variation in females. On the other hand, we do not expect a correlation between allonursing behaviour and annual variation in body mass in females.

## Methods

### CV body mass as a proxy for the tendency to store body fat

In mammals, body fat explained between 41 and 92% of the intraspecific variation in body mass, the amount of body fat was highly correlated with carcass weight for each age and sex; hence body weight was a good predictor of total body fat (for a summary, see references in [44]). We therefore used seasonal changes of body mass over a year as a proxy for the tendency to store body fat. For a given species, we calculated the coefficient of variation (CV = standard deviation/mean) over monthly means of adult female body mass, yielding a total sample of 87 mammalian species from 9 orders (Additional files 1 and 2). In a previous study we validated the use of CV body mass as a proxy for variation in body fat by showing that the monthly body mass correlated with percentage body fat in several studies that measured both in the same specimens [44].

We compiled monthly body mass data from the literature, including only those studies that reported monthly mean body mass for at least 4 months per year. If body mass data were given for four seasons, pooled across several months (e.g., spring, summer, autumn and winter), we set the number of months sampled to four (16 studies). In most species, monthly mean body mass data was distributed evenly across the year, except for *Antechinus stuartii*, *Lycaon pictus*, *Spermophilus franklinii* and *Zapus hudsonicus*. If several sources were available for one species, preference was given to the study with the largest sample size conducted in the wild.

### Allomaternal care behaviours

In quantifying allomaternal care behaviour, we followed Isler and van Schaik [76] to obtain continuous data on the frequency of occurrence of the following care behaviours: provisioning, carrying, protection and a variable that comprises other energetically influential care behaviours such as huddling, communal nesting and pup retrieval (see Additional file 3 for a detailed description of the classification protocol). As the sample in [76] was restricted to species with known brain size, we expanded it by an additional 30 species for which data on both CV body mass and allomaternal care behaviour was available in the literature (Additional files 1 and 2). In total, CV body mass and data on allomaternal care behaviour were available for 87 species. We did not compile data for bats and cetaceans because reliable data on allomaternal care of both cetaceans and bats are notoriously difficult to obtain. Moreover, the amount of body fat and hence CV body mass as a proxy for the tendency to store body fat in these two groups may underlie different constraints than in other mammals [44, 77–79], precluding predictions for a combined sample.

In addition, to distinguish the effects of allomaternal care provided by males (paternal care) from that provided by other group members (care by others) we summed up the frequency of occurrence of all allomaternal care behaviours separately for the father and other group members. To investigate whether the results reported in this study are robust with respect to different coding schemes of allomaternal care, we additionally conducted all analyses by using a binary classification of all allomaternal care behaviours, with 1 indicating the presence and 0 the absence of the helping behaviour. Finally, we also conducted additional analyses with a binary classification of allomaternal care provided by males (paternal care) and that provided by other group members (care by others) (data from [76, 80, 81]).

### Covariates

As captivity might affect body mass variation (for instance, under good husbandry conditions, most animals gain weight in captivity [82]), we added provenance (wild = 1/captivity = 0) as an additional factor in all analyses. Furthermore, we analysed the subsample of studies including only wild-caught females separately.

In a previous study we found that substrate use (arboreal versus terrestrial) influenced the amount of body fat of a species [44]. We therefore added substrate use as an additional factor in all analyses. Data from published sources were used to assign each species to one of two substrate use categories, terrestrial (0) or arboreal (1), based on their main habit. Species were classified as terrestrial when they spent more than 50% of observation time on the ground ([83–86], see Additional file 1).

We also controlled for several other potential methodological confounds. First, some studies include body mass data from pregnant and lactating females in the population mean, which may artificially increase annual body mass variation in seasonal breeders. Pregnancy affects a female’s weight due to the added weight of the offspring and the associated tissues and fluids. To control for this effect, we added the variable "inclusion of reproductive females in the study" as a covariate. Second, we added the number of months sampled as covariate. Ideally, we would have preferred to use only those studies from the wild that reported the mean body mass for 12 consecutive months. However, in contrast to studies in captivity, most body mass data of wild living mammals have been recorded less frequently. Third, to control for allometric effects of size, we performed all analyses including log-transformed mean body mass as a covariate, taking the overall mean from the same specimens for which CV body mass was determined. Finally, as variation in female body mass may be influenced by life history traits such as litter size, neonatal mass, and the duration of gestation and lactation, we also included those as potential covariates.

### Statistical analyses

Statistical analyses were done in JMP™ 12.0 [87] and in R3.1.3 [88]. In most species that exhibit allomaternal care, various kinds of care behaviours are observed, potentially resulting in collinearity problems in the statistical analyses. We checked this by generating variance inflation factors (VIF) to assess potential multicollinearity in the full set of allomaternal care behaviours [89, 90] using non-phylogenetic generalized linear models and the function “vif” (“car” package: [91]) in R. VIFs quantify how much the variance of an estimated model parameter is increased because of multicollinearity between predictors. The VIF for carry by the male, carry by others, provisioning by the male and provisioning by others was higher than 5, which indicates a problematic amount of covariance among predictors [92]. To solve this, we summed up the frequency of occurrence of carrying by the male and by others to one single variable “carrying” and similarly provisioning by the male and provisioning by others to “provisioning”. After this, the VIF of all allomaternal care behaviours in all models were less than 4, which indicates an acceptable amount of covariance among predictors (Additional file 4: Tables S1 and S2). Two life history traits (duration of gestation and neonatal mass) also showed VIFs consistently larger than 5 in all models (Additional file 4: Tables S1 and S2). To reduce the problematic multicollinearity in these models, we followed the method described in [93]: we first removed the life history variable with the highest VIF value from the models, the duration of gestation, and recalculated VIFs for the reduced models. Then, we removed neonatal mass, as it still had a VIF larger than 5. All remaining variables had VIFs lower than 5. We then repeated the analyses with the same specifications as the main analysis with these “reduced models” and assessed the relative contribution of each independent variable as described below.

We built phylogenetic generalized least-squares regressions (PGLS) models [94, 95] using the “caper” package [96] in R. Caper estimates PGLS model parameters in maximum likelihood [96] and the parameter lambda (λ), which quantifies the magnitude of the phylogenetic signal in the model residuals [94]. The value of λ can vary between 0, indicating no phylogenetic signal, and 1, indicating that the observed pattern fits a Brownian motion model of trait evolution along the branches of the phylogeny such that similarity between species is directly proportional to relatedness [94]. The phylogeny was based on a composite supertree from [97] (Additional file 5: Figure S1). CV body mass (used as a proxy for body fat) was the dependent variable, while measures of allomaternal care and all possible confounding variables (substrate use, provenance [wild / captivity], number of months sampled, inclusion of reproductive females, mean body mass and several life history variables) were independent variables in the PGLS models. We did not log-transform CV body mass values prior to the analysis as this would not have improved the skew of its distribution. Although the predictor CV body mass was skewed towards smaller values, the distribution of the residuals of the PGLS models were normally distributed and did not comprise any outliers.

We used a model selection approach based on the AICc (Aikaike Information Criterion with correction for finite sample size, [98]) to determine the most important allomaternal care behaviours for female CV body mass. We ran the model selection across all possible models built with the explanatory variables mentioned above. We accounted for uncertainty in the models by performing model averaging [99] in the candidate model set including models with ∆AICc <2 [100]. ∆AICc is the difference in AICc between the focal model and the AICc of the best-fitting model in the candidate model set. Estimates of each parameter were averaged across the candidate models (means were weighted by the Akaike weight of a given model). The relative importance of a predictor was obtained by summing the Akaike’s weights of the models in the candidate model set including the focal predictor, following the method described by Symonds and Moussalli [101]. The method to perform model averaging with the PGLS function in the package “caper” [96] is described in [102] and the corresponding material is available at http://www.mpcm-evolution.org.

## Results

**Fig. 1:**
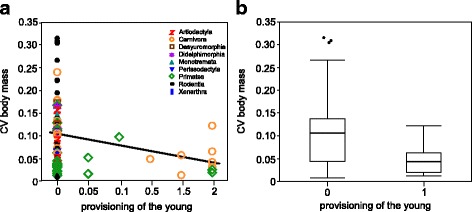
**a** Female CV body mass as a function of provisioning of the young by the male and other group members, using the continuous coding scheme. **b** Female CV body mass is lower in species with provisioning of the young by the male and other group members (coded as 1) than in species without it (coded as 0). Details of phylogenetic models are shown in Table 1 and Additional file 4: Table S6. Species values are listed in the Additional file 1

The results confirmed our two main predictions. Model selection and averaging showed that the most important effect among allomaternal care behaviours on female CV body mass was provisioning of the young by the male and other group-members (Relative importance = 1) (Table 1, Fig. 1a). This form of allomaternal care was negatively correlated with CV body mass in reproductive females, suggesting that an energetic contribution towards offspring rearing allows females to reduce the amount of stored body fat. In contrast, allonursing, which involves no additional influx of energy but distributes maternal help across different mothers, did not correlate with CV body mass (Relative importance = 0.06) (Table 1). Results using a binary coding scheme of allomaternal care behaviours are strikingly similar (Additional file 4: Table S6 and S8, Fig. 1b).

**Table 1:** Continuous classification of allomaternal care behaviours: Averaged parameter estimates and their relative explanatory importance for female CV body mass (*N = 87*). Gestation length and neonatal mass were excluded to reduce multicollinearity between predictors. Numbers in bold indicate predictors whose confidence intervals of their effect exclude zero

Predictors		Relative importance of predictors	Model averaging estimates^a^	95% CI
Intercept			0.126	**(0.100, 0.153)**
Provisioning		1.00	−0.040	**(−0.043, −0.036)**
Protecting		0.06	−0.001	(−0.002, 0.001)
Carrying		0.07	0.003	(−0.004, 0.010)
Communal nesting		0.06	0.001	(−0.002, 0.004)
Allonursing		0.06	0.005	(−0.010, 0.021)
Log mean body mass		0.44	−0.006	**(−0.010, −0.002)**
Provenance	captive	0.80	na	na
	wild		0.025	**(0.017, 0.032)**
Substrate use	terrestrial	1.00	na	na
	arboreal		−0.045	**(−0.050, −0.041)**
Number of months		0.53	−0.001	(−0.001, 0.001)
Inclusion of reproductive females		0.69	−0.019	**(−0.030, −0.008)**
Log litter size		0.56	0.027	**(0.013, 0.041)**
Log weaning age		na	0	0

^a^averaged model estimates based on 12 models with ΔAICc (AICc _focal model_ – AICc _best model_) < 2 since the best AICc model is not strongly weighted (weight = 0.15) [104]. A full list of models is given in Additional file 4: Table S4. Reference levels of categorical variables have an estimate of 0; na – not applicable; 95% CI - 95% confidence interval

**Fig. 2:**
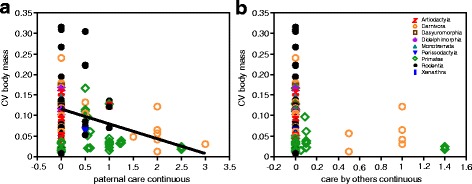
Female CV body mass is lower in species with paternal care **a** but not with care provided by other group members (**b**), using the continuous coding scheme. Details of phylogenetic models are shown in Table 2. Species values are listed in the Additional file 1

Using a continuous coding scheme of paternal care and the amount of allomaternal care provided by other group members, we found that only paternal care showed a negative relationship with CV body mass (Relative importance = 1) (Table 2, Fig. 2a and b). In contrast, using a binary coding scheme, both paternal care and the amount of allomaternal care provided by other group members had a negative effect on CV body mass, although the negative effect of paternal care was stronger than that of allomaternal care by other group members (Additional file 4: Table S7 and S9, Figure S2a and b).

**Table 2:** Continuous classification of paternal care and care provided by other group members: Averaged parameter estimates and their relative explanatory importance for female CV body mass (*N* = 87). Gestation length and neonatal mass were excluded to reduce multicollinearity between predictors. Numbers in bold indicate predictors whose confidence intervals of their effect exclude zero

Predictors		Relative importance of predictors	Model averaging estimates^a^	95% CI
Intercept			0.148	**(0.127, 0.169)**
Care by others		na	0	0
Paternal care		1.00	−0.028	**(−0.029, −0.027)**
Log mean body mass		0.67	−0.008	**(−0.011, −0.004)**
Provenance	captive	0.80	na	na
	wild		0.024	**(0.017, 0.032)**
Substrate use	terrestrial	1.00	Na	na
	arboreal		−0.047	**(−0.050, −0.043)**
Number of months		0.38	−0.001	**(−0.002, −0.001)**
Inclusion of reproductive females		0.37	−0.011	**(−0.020, −0.002)**
Log litter size		0.24	0.007	(−0.001, 0.016)
Log weaning age		na	0	0

^a^averaged model estimates based on 11 models with ΔAICc (AICc _focal model_ – AICc _best model_) < 2 since the best AICc model is not strongly weighted (weight = 0.15) [104]. A full list of models is given in Additional file 4: Table S5. Reference levels of categorical variables have an estimate of 0; na – not applicable; 95% CI - 95% confidence interval

Results for the subset of studies including only wild-caught females (*N* = 49 species) were largely similar to those obtained from the whole sample, although the effects were a bit weaker (Additional file 4: Tables S10-S15).

In all analyses substrate use and provenance were correlated with CV body mass. Arboreal species had less body fat than terrestrial and semiaquatic species, as indicated by the negative correlation between CV body mass and substrate use. Furthermore, CV body mass was higher in wild-caught specimens compared to captive ones, suggesting that wild-caught individuals experience more variation in energy intake than provisioned specimens living in captivity. Controlling for further possible confounding variables (number of months sampled, inclusion of reproductive females, mean body mass, and several life history variables) did not change the effects of the main explanatory variables. In some models, both a lower species body mass and the inclusion of reproductive females in the study were related to a lower CV body mass, while species with a relatively high reproductive rate, as indicated by larger litters, exhibited a higher CV body mass. In some models, species for which fewer months were sampled showed a larger CV body mass (Tables 1 and 2 and Additional file 4: Tables S6, S7, S12 and S13).

## Discussion

Using annual variation in body mass, we found that this CV body mass and the amount of allomaternal care show a pattern of correlated evolution among female mammals: females of those species with more contributions of non-mothers to offspring care exhibit reduced annual variation in body mass. From this, we conclude that allomaternal energy subsidies and fat storage are compensatory strategies to stabilise the energetic costs involved in female reproduction.

First, we predicted that only an additional influx of energy in the form of costly allomaternal care behaviours by the male and other non-breeding group members towards the offspring and the mother would allow reproductive females to reduce the storage of body fat, whereas a mere redistribution of energy between mothers as in allonursing behaviour would not. As predicted, we only found a negative correlation between seasonal variation in body mass and the amount of allomaternal care in the form of provisioning of the young by the male and other group members, but not with allonursing. This suggests that if other conspecifics take over some of the maternal costs the need for these females to store extra body fat to fuel reproduction is relaxed.

This pattern across species is consistent with numerous intraspecific studies showing that extra energy delivered by costly care behaviours of helpers allows breeding females to reduce their maternal investment. For instance, in meerkats and cooperatively breeding bird species, an increased number of helpers enabled breeding females to maintain better condition and higher body mass and achieve a higher fitness [103–107]. In Campbell’s dwarf hamsters (*Phodopus campbelli*) the presence of males protects females against extreme heat production in response to the exogenous heat requirements of the pups. As this acute increase in maternal temperature is thought to be a substantial cost to females, paternal presence likely allows females to decrease the energetic demands of reproduction [108]. Another study of the same species found that removal of the male not only decreased pup survival, growth, and readiness for dispersal by 18 days of age but also resulted in an additional 20% body weight loss in the female [109]. Lastly, a comparative study across mammals reveals that male care is associated with larger litters in some species or shorter lactation time in others, resulting in increased female fecundity [51].

Second, we investigated the effect of different types of allomaternal help (help of the male or other conspecifics) on female fat stores. Both the help provided by the breeding male and the help provided by other group members showed a negative correlation with female CV body mass. However, the relative importance of allomaternal care provided by the breeding male was greater than the relative importance of help of other caretakers. This fits well with the often-reported finding that males care unconditionally, whereas care by helpers may be more conditional [71, 72, 110].

A broad comparative study as presented here can only provide an overview over potential patterns of correlated evolution and is limited by methodological issues. Ideally, we would have preferred to use individual variation in body fat over the year instead of the annual variation in body mass averaged over several females as used in this study. Although the published literature contains a variety of measures of adipose depots in living subjects such as palpation, skinfold thickness, perirenal adiposity, the number of adipocytes in bone marrow, and adipocyte volumes from tissue samples [111], these measures have not yet been compared to each other and each measure has only been applied to very few different species making broad phylogenetic comparisons impossible. Similarly, taking body fat values obtained from cadavers is problematic because they assess body fat at a single point in time, while the individual body fat fluctuations remain unknown [73].

It may be argued that, rather than taking annual variation in body mass, the costs of reproduction should be estimated by subtracting the maternal body weight at conception from the body weight at offspring weaning. However, such detailed data are rarely available, and may raise other issues, such as postpartum oestrus in lagomorphs, Callitrichid primates and several otariids, which means females suckle newborns while simultaneously being pregnant [112–115]. Even more importantly, in most mammals such as carnivores, rodents and primates allomaternal care and its beneficial effect for mothers continues post-weaning. Thus, offspring provisioning until independence allows females to invest more time in foraging, regain body condition more quickly and mate sooner [116], which we would not capture with the body weight difference of mothers between conception and offspring weaning.

In our study, some part of the variation in female body mass may result from the increasing weight of the foetus or litter during gestation. However, without dissection this cannot be disentangled from storing energy reserves during gestation for the subsequent lactation period, which is even more energetically demanding [33]. As a rough control for such effects, we included neonatal mass, litter size, gestation length and lactation time as potential correlates in the analyses, but this did not alter our findings. Moreover, because cooperative breeders tend to have higher reproductive efforts than independent breeder [117], this possibility cannot explain the reduced CV in body mass among species receiving allomaternal care.

In our data, we expect a relatively weak phylogenetic signal of CV body mass and thus low values of λ as the amount of body fat is phenotypically plastic and can undergo quick and extensive adaptive modifications in response to food availability and local environment. Therefore, closely related species might have very different CV body masses depending on their habitats [118–120].

Another unsolved question concerns the relationship between reproductive effort, seasonal fluctuations in climate or food abundance, and social factors such as allomaternal care. Reproductive seasons and experienced seasonality in food intake are generally interrelated in mammals [30]. There is evidence that species inhabiting more seasonal and less predictable habitats more often breed cooperatively [121, 122], and we also expect that they would benefit more from a higher ability to store body fat. However, because we found a negative, rather than the expected positive correlation between allomaternal care and the tendency to store body fat, this confirms that there is indeed a trade-off due to energetic costs of fat storage, and thus that social and physiological buffers are compensatory strategies to maintain fitness in a harsh environment. To further investigate these strategies, we would not only need data on environmental factors such as annual rainfall, vegetation indices or actual food abundance, but also of the seasonality experienced by the animals themselves, as expressed in dietary habits throughout the year, analogous to our studies of brain size and seasonality in primates [123–125].

## Conclusions

In conclusion, several lines of evidence suggest that any allomaternal care, be it aimed at the mother or the offspring, and be it by the father or other conspecifics, allows females to reduce the amount of stored body fat. In combination with intraspecific studies, our results further support the idea that the main reason for this negative correlation between the amount of allomaternal care and female CV body mass is the energetic contribution towards offspring rearing through costly care by males or helpers, which stabilises the energetic costs for female reproduction. Although our comparative approach has some limitations, our analyses indicate that female mammals have two different strategies of coping with energetic constraints on reproduction: either getting fat or getting help.

## Additional files

Additional file 1: List of species and data used for this study. (XLSX 22 kb)

Additional file 2: References for the CV body mass data used for this study. (DOCX 60 kb)

Additional file 3: Compilation and quantification of allomaternal care behaviours. (DOCX 37 kb)

Additional file 4: Supplementary results:** Tables S1 and S2.** Results testing for collinearity among predictors. Variation inflation factors (VIF) for all the full models and all the reduced models after multicollinearity is considered. **Table S3.** Estimated phylogenetic signal (λ) in the individual variables. **Tables S4 and S5.** Model sets obtained after model selection based on ΔAICc <2 including best-supported models and multiple-model parameter estimates. **Tables S6-S9 and Figure S2.** Results of a binary coding scheme of allomaternal care behaviours as well as binary coded care provided by males (paternal care) or other group members (care by others). **Tables S10-S15.** Results for the subset of studies including only wild-caught females (*N* = 49). These remained largely identical to those obtained with the whole dataset (see also Tables 1 and 2 in the main text). (DOCX 163 kb)

Additional file 5: Figure S1. Phylogenetic tree of 87 mammal species used in this study visualised using Mesquite v. 3.11 [126]. (PDF 83 kb)
