# Correction to: getting fat or getting help? how female mammals cope with energetic constraints on reproduction

**DOI:** 10.1186/s12983-017-0236-7

**Published:** 2018-02-08

**Authors:** Sandra A. Heldstab, Carel P. van Schaik, Karin Isler

**Affiliations:** Department of Anthropology, University of Zurich, Winterthurerstrasse 190, 8057 Zurich, Switzerland

## Correction

Unfortunately, upon publication of this article [1] it was noticed there was an error within the Discussion section. During proofing a request to edit the following paragraph was missed:

“In our data, we expect a relatively weak phylogenetic signal of CV body mass and thus low values of λ as the amount of body fat is phenotypically plastic and can undergo quick and extensive adaptive modifications in response to food availability and local environment. Therefore, closely related species might have very different CV body masses depending on their habitats [118, 119, 120].”

This should read:

“In our data, we found a surprisingly weak phylogenetic signal of CV body mass and thus low values of λ for the model residuals, indicating that the phylogenetic disposition for fat disposition is partially masked by habitat-caused variation [118, 119, 120]. The fact that we still found significant relationships between CV body mass and allomaternal care would then make our case even stronger, because it implies that the underlying effect must be very strong.”

The original article has now been updated and the publisher apologizes for any inconvenience caused.
