# Supplementary material for: Getting fat or getting help? How female mammals cope with energetic constraints on reproduction
Source: Front Zool. 2017 Jun 12;14:29. doi: 10.1186/s12983-017-0214-0 (PMC5468974; doi:10.1186/s12983-017-0214-0)
Supplement: Supplementary file 2 — References for the CV body mass data used for this study. (DOCX 60 kb) [file 12983_2017_214_MOESM2_ESM.docx]

**Supplementary references**

1. Franzmann AW, Arneson PD. Marrow fat in Alaskan moose femurs in relation to mortality factors. J Wildl Manage 1976;40:336-339.

2. Weber ML, Thompson JM. Seasonal patterns in food intake, live mass, and body composition of mature female fallow deer (*Dama dama*). Can J Zool 1998;76:1141-1152.

3. Adamczewski JZ, Flood PF, Gunn A. Seasonal patterns in body composition and reproduction of female muskoxen (*Ovibos moschatus*). J Zool 1997;241:245-269.

4. Chan-McLeod ACA, White RG, Russell DE. Comparative body composition strategies of breeding and nonbreeding female caribou. Can J Zool 2000;77:1901-1907.

5. Skinner JD. Productivity of mountain reedbuck *Redunca fulvorufula* (Afzelius, 1815) at the Mountain Zebra National Park. Koedoe 1980;23:123-130.

6. Schaschl H, Suchentrunk F, Morris DL, Slimen HB, Smith S, Arnold W. Sex-specific selection for MHC variability in Alpine chamois. BMC Evol Biol 2012;12:20-30.

7. Seal U, Mech L. Blood indicators of seasonal metabolic patterns in captive adult gray wolves. J Wildl Manage 1983;47:704-715.

8. Beck CA, Bowen WD, Iverson SJ. Sex differences in the seasonal patterns of energy storage and expenditure in a phocid seal. J Anim Ecol 2003;72:280-291.

9. Visee A. African wild dog (*Lycaon pictus*) breeding programme: the Wildlife Preservation Trust Fund (Tanzania), the George Adamson Wildlife Preservation Trust (UK, USA, GER.), the African Wild Dog Foundation (NETH). Report 1995-2001, Mkomazi Game Reserve, Tanzania 2001.

10. Korhonen H, Harri M. Seasonal changes in energy economy of farmed polecat as evaluated by body weight, food intake and behavioural strategy. Physiol Behav 1986;37:777-783.

11. Moore DW, Kennedy ML. Weight changes and population structure of racoons in Western Tennessee. J Wildl Manage 1985;49:906-909.

12. Crabb WD. Growth, development and seasonal weights of spotted skunks. J Mammal 1944;25:213-221.

13. Sullivan EG. Gray fox reproduction, denning, range, and weights in Alabama. J Mammal 1956;37:346-351.

14. Hashimoto Y, Yasutake A. Seasonal changes in body weight of female Asiatic black bears under captivity. Mamm Study 1999;24:1-6.

15. Prestrud P, Nilssen K. Fat deposition and seasonal variation in body composition of Arctic foxes in Svalbard. J Wildl Manage 1992;56:221-233.

16. Banks PB, Dickman CR. Effects of winter food supplementation on reproduction, body mass, and numbers of small mammals in montane Australia. Can J Zool 2000;78:1775-1783.

17. Fleming PA, Nicolson SW. Arthropod fauna of mammal-pollinated *Protea humiflora*: ants as an attractant for insectivore pollinators? Afr Entomol 2003;11:9-14.

18. Fleming PA, Nicolson SW. Opportunistic breeding in the Cape spiny mouse (*Acomys subspinosus*). Afr Zool 2002;37:101-105.

19. Nicol S, Andersen NA. The life history of an egg-laying mammal, the echidna (*Tachyglossus aculeatus*). Ecoscience 2007;14:275-285.

20. Scheibe KM, Streich WJ. Annual rhythm of body weight in Przewalski horses (*Equus ferus przewalskii*). Biol Rhythm Res 2003;34:383-395.

21. Mueller AE. Aspects of social life in the fat-tailed dwarf lemur (*Cheirogaleus medius*): inferences from body weights and trapping data. Am J Primatol 1999;49:265-280.

22. Zehr SM, Roach RG, Haring D, Taylor J, Cameron FH, Yoder AD. Life history profiles for 27 strepsirrhine primate taxa generated using captive data from the Duke Lemur Center. Sci Data 2014;1:1-11.

23. Zehr SM, Roach RG, Haring D, Taylor J, Cameron FH, Yoder AD. Data from: Life history profiles for 27 strepsirrhine primate taxa generated using captive data from the Duke Lemur Center. Dryad Digital Repository. (http://dx.doi.org/10.5061/dryad.fj974). 2014.

24. Dietz JM, Baker AJ, Miglioretti D. Seasonal variation in reproduction, juvenile growth, and adult body mass in golden lion tamarins (*Leontopithecus rosalia*). Am J Primatol 1994;34:115-132.

25. Muroyama Y, Kanamori H, Kitahara E. Seasonal variation and sex differences in the nutritional status in two local populations of wild Japanese macaques. Primates 2006;47:355-364.

26. Randrianambinina B, Rakotondravony D, Radespiel U, Zimmermann E. Seasonal changes in general activity, body mass and reproduction of two small nocturnal primates: a comparison of the golden brown mouse lemur (*Microcebus ravelobensis*) in Northwestern Madagascar and the brown mouse lemur (*Microcebus rufus*) in Eastern Madagascar. Primates 2003;44:321-331.

27. Uehara S, Nishida T. Body weights of wild chimpanzees (*Pan troglodytes schweinfurthii*) of the Mahale Mountains National Park, Tanzania. Am J Phys Anthropol 1987;72:315-321.

28. Lewis RJ, Kappeler PM. Seasonality, body condition, and timing of reproduction in *Propithecus verreauxi verreauxi* in the Kirindy Forest. Am J Primatol 2005;67:347-364.

29. Del Valle JC, Busch C. Body composition and gut length of *Akodon azarae* (Muridae: Sigmodontinae): relationship with energetic requirements. Acta Theriol 2003;48:347-357.

30. Tileston JV, Lechleitner R. Some comparisons of the black-tailed and white-tailed prairie dogs in north-central Colorado. Am Nat 1966;75:292-316.

31. Sweitzer RA, Berger J. Seasonal dynamics of mass and body condition in Great Basin porcupines (*Erethizon dorsatum*). J Mammal 1993;74:198-203.

32. Zatzman ML, Thornhill GV, Ray WJ, Ellersiek MR. Seasonal changes of food and water consumption and urine production of the marmot, *Marmota flaviventris*. Comp Biochem Physiol A 1984;77:735-743.

33. Concannon P, Levac K, Rawson R, Tennant B, Bensadoun A. Seasonal changes in serum leptin, food intake, and body weight in photoentrained woodchucks. Am J Physiol-Reg I 2001;281:R951-R959.

34. Zhang Z-Q, Wang D-H. Seasonal changes in thermogenesis and body mass in wild Mongolian gerbils (*Meriones unguiculatus*). Comp Biochem Physiol A 2007;148:346-353.

35. Batzli GO, Pitelka FA. Condition and diet of cycling populations of the California vole, *Microtus californicus*. J Mammal 1971;52:141-163.

36. Voltura MB. Seasonal variation in body composition and gut capacity of the prairie vole (*Microtus ochrogaster*). Can J Zool 1997;75:1714-1719.

37. Iverson SL, Turner BN. Seasonal variation in body composition of the meadow vole (*Microtus pennsylvanicus*). In: Wali MK, editor. Prairie: A Multiple View. North Dakota: University of North Dakota Press, Grand Forks; 1975. p. 133-141.

38. Lochmiller RL, Whelan JB, Kirkpatrick RL. Body composition and reserves of energy of *Microtus pinetorum* from Southwest Virginia. Am Nat 1983;110:138-144.

39. Zuercher GL, Roby DD, Rexstad EA. Seasonal changes in body mass, composition, and organs of northern red-backed voles in interior Alaska. J Mammal 1999;80:443-459.

40. Cameron GN, Spencer SR. Field growth-rates and dynamics of body mass for rodents on the Texas Coastal Prairie. J Mammal 1983;64:656-665.

41. Short HL, Duke WB. Seasonal food consumption and body weights of captive tree squirrels. J Wildl Manage 1971;35:435-439.

42. Lurz PWW, Lloyd AJ. Body weights in grey and red squirrels: do seasonal weight increases occur in conifer woodland? J Zool 2000;252:539-543.

43. Choromanskinorris J, Fritzell EK, Sargeant AB. Seasonal activity cycle and weight changes of the Franklin's ground squirrel. Am Nat 1986;116:101-107.

44. Blake BH. Annual cycle and fat storage in 2 populations of golden mantled ground squirrels. J Mammal 1972;53:157-167.

45. Buck CL, Barnes BM. Annual cycle of body composition and hibernation in free-living arctic ground squirrels. J Mammal 1999;80:430-442.

46. Koprowski JL. Annual cycles in body mass and reproduction of endangered Mt. Graham red squirrels. J Mammal 2005;86:309-313.

47. Morrison P, Ryser FA. Metabolism and body temperature in a small hibernator, meadow jumping mouse, *Zapus hudsonicus*. J Cell Comp Physiol 1962;60:169-180.

48. Silva SM, Summa JL, Summa MEL, Geraldi VC, Belluci M, Klefasz A, Morgante JS, Moraes-Barros N. Contribution of wildlife governmental centers to conservation and biological study of sloths *Bradypus variegatus*. Nat Conserv 2014;12:79-85.

49. Dunstone N, Davies J. The Mink. London, UK: Poyser Natural History; 1993.

50. Fairley JS. The food, reproduction, form, growth and development of the fox *Vulpes vulpes* (L.) in north-east Ireland. Proc R Ir Acad B 1970;69:103-137.

51. Poulle M-L, Crete M, Huot J. Seasonal variation in body mass and composition of eastern coyotes. Can J Zool 1995;73:1625-1633.

52. Verts B. Biology of the Striped Skunk. Urbana, IL: University of Illinois Press; 1967.

53. Carstairs J. Seasonal changes in organ weights of *Rattus villosissimus* during the 1966-69 'Plague'at Brunette Downs, Northern Territory. Aust J Zool 1980;28:173-183.

54. Michener GR. Effect of age and parity on weight gain and entry into hibernation in Richardson's ground squirrels. Can J Zool 1978;56:2573-2577.

55. Morton SR. An ecological study of *Sminthopsis crassicaudata* (Marsupialia: Dasyuridae) III.* Reproduction and life history. Wildl Res 1978;5:183-211.

56. Khokhlova I, Degen AA, Krasnov BR, Shenbrot GI. Body mass and environment: a study in Negev rodents. Israel J Zool 2001;47:1-13.

57. Warrick GD, Cypher BL. Variation in body mass of San Joaquin kit foxes. J Mammal 1999;80:972-979.
