# Supplementary material for: Getting fat or getting help? How female mammals cope with energetic constraints on reproduction
Source: Front Zool. 2017 Jun 12;14:29. doi: 10.1186/s12983-017-0214-0 (PMC5468974; doi:10.1186/s12983-017-0214-0)
Supplement: Supplementary file 3 — Compilation and quantification of allomaternal care behaviours. (DOCX 37 kb) [file 12983_2017_214_MOESM3_ESM.docx]

**Compilation and quantification of allomaternal care behaviours**

Data for this study was retrieved from [[1](#_ENREF_1)] and supplemented with additional data for 30 species collected in the same manner. Data on allomaternal care behaviours were compiled and quantified as follows:

Values on allomaternal care behaviour were compiled from published compilations [[2-17](#_ENREF_2)], the Mammalian Species accounts (1969-2014), reliable online sources (Animal Diversity Web [[18](#_ENREF_18)], and All the World’s Primates [[19](#_ENREF_19)] and original sources for individual species (see also [[1](#_ENREF_1)])).

Allomaternal care behaviour was divided into the following categories: carrying, provisioning, allonursing, protection, thermoregulation/babysitting and pup retrieval and coded as follows:

**Provisioning by the male/provisioning by others:** This refers to the frequency of provisioning by the male/by other group members. It was set to 1, if the male/other group members usually actively shared or provisioned food, to 0.75 if passive sharing was more frequent (70-80%) but active sharing was common (20-30%), to 0.5 if passive sharing was common and active sharing rare, to 0.1 if passive sharing was rare, and to 0.05 if single observations of food sharing or provisioning were reported.

**Carrying by the male/carry by others:** This refers to the frequency of carrying by the male/by other group members. If the offspring was carried 50% of the time, the value was 0.5. When carrying behaviour was limited to pup retrieval it was counted in the category of thermoregulation, babysitting and pup retrieval below.

**Protection:** This refers to the occurrence of active protection by the male, defence of territory, or defence against predators. It was considered absent if territories were only protected against other males, and females or young may even be hurt during agonistic encounters between males. Otherwise, we scored protection as 1 if it was usual, 0.5 if frequent, 0.1 if rare, and 0.05 if single observations were reported. If more precise values were given in the original sources, those were used.

**Communal nesting, babysitting and pup retrieval:** This refers to the occurrence of babysitting during the mother’s absence, retrieving pups, or carrying offspring to a new nest, by the male or other group members, huddling, and communal nesting during the breeding period. It was scored as follows: 1 if it was usual, 0.5 if frequent, 0.1 if rare, and 0.05 if single observations were reported. If more precise values were given in the original sources, those were used.

**Allonursing:** This refers to the frequency of allonursing of an infant. The values given by [[8](#_ENREF_8)] were converted as follows: (<10%) was set to 0.1, 10-45% (less than own mother) to 0.25, and as much as own mother to 0.5. If more precise values were given in the original source, these were used.

**References**

1. Isler K, van Schaik CP. Allomaternal care, life history and brain size evolution in mammals. J Hum Evol 2012;63:52-63.

2. Spencer-Booth Y. The relationships between mammalian young and conspecifics other than mothers and peers: a review. Adv Study Behav 1971;3:119-194.

3. Gubernick DJ. Parent and infant attachment in mammals. In: Gubernick DJ, Klopfer P, editors. Parental Care in Mammals. 10.1007/978-1-4613-3150-6_7. New York, NY: Plenum Press; 1981. p. 243-305.

4. Dewsbury DA. Paternal behavior in rodents. Am Zool 1985;25:841-852.

5. Whitten PL. Infants and adult males. In: BB S, Cheney D, Seyfarth R, Wrangham R, Struhsaker T, editors. Primate Societies. Chicago, IL: University of Chicago Press; 1987. p. 343-357.

6. Bronson FH. Mammalian Reproductive Biology. Chicago, IL: University of Chicago Press; 1989.

7. Emlen ST. Cooperative Breeding in Birds and Mammals. Oxford: Blackwell Scientific Publications; 1984.

8. Packer C, Lewis S, Pusey A. A comparative analysis of non-offspring nursing. Anim Behav 1992;43:265-281.

9. Woodroffe R, Vincent A. Mother's little helpers: patterns of male care in mammals. Trends Ecol Evol 1994;9:294-297.

10. Snowdon CT. Infant care in cooperatively breeding species. Adv Stud Behav 1996;25:643-689.

11. Hayes LD. To nest communally or not to nest communally: a review of rodent communal nesting and nursing. Anim Behav 2000;59:677-688.

12. Ross C, MacLarnon A. The evolution of non-maternal care in anthropoid primates: a test of the hypotheses. Folia Primatol 2000;71:93-113.

13. Silk JB. The adaptive value of sociality in mammalian groups. Proc R Soc B 2007;362:539-559.

14. Wilson DE, Mittermeier RA. Handbook of the Mammals of the World. Barcelona, ES: Lynx Edicions; 2009.

15. König B. Non-offspring nursing in mammals: general implications from a case study on house mice. In: Kappeler PM, van Schaik CP, editors. Cooperation in Primates and Humans. 10.1007/3-540-28277-7_11. Berlin, DE: Springer; 2006. p. 191-205.

16. Gittleman JL. Female brain size and parental care in carnivores. Proc Natl Acad Sci USA 1994;91:5495-5497.

17. Solomon NG, French JA. The study of mammalian cooperative breeding. Cooperative Breeding in Mammals. Cambridge, UK: Cambridge University Press; 1997. p. 1-10.

18. The Animal Diversity Web. Myers P, Espinosa R, Parr C, Jones T, Hammond G, Dewey T. 2006. http://animaldiversity.org/accounts/Mammalia/. Accessed 5 Sept 2016.

19. All the World's Primates. Rowe N, Myers M. 2011. http://www.alltheworldsprimates.org. Accessed 5 Sept 2016.
