# Supplementary material for: Getting fat or getting help? How female mammals cope with energetic constraints on reproduction
Source: Front Zool. 2017 Jun 12;14:29. doi: 10.1186/s12983-017-0214-0 (PMC5468974; doi:10.1186/s12983-017-0214-0)
Supplement: Supplementary file 4 — Supplementary results: Tables S1 and S2. Results testing for collinearity among predictors. Variation inflation factors (VIF) for all the full models and all the reduced models after multicollinearity is considered. Table S3. Estimated phylogenetic signal (λ) in the individual variables. Tables S4 and S5. Model sets obtained after model selection based on ΔAICc <2 including best-supported models and multiple-model parameter estimates. Tables S6-S9 and Figure S2. Results of a binary coding scheme of allomaternal care behaviours as well as binary coded care provided by males (paternal care) or other group members (care by others). Tables S10-S15. Results for the subset of studies including only wild-caught females (N = 49). These remained largely identical to those obtained with the whole dataset (see also Tables 1 and 2 in the main text). (DOCX 163 kb) [file 12983_2017_214_MOESM4_ESM.docx]

**Supplementary results**

***Results testing for collinearity among predictors***

**Table S1.** Variance inflation factors (VIF) of the predictor variables for female CV body mass in the full models and the reduced models, when all allomaternal care behaviours are entered separately as predictors. Values of VIF greater than 5 indicate a problematic amount of covariance among predictors [[1](#_ENREF_1)]. For the reduced models “na” indicate that a given predictor variable has been excluded from the models due to its high VIF value in the full model.

|  | **continuous classification of allomaternal care behaviours** | |  | **binary classification of allomaternal care behaviours** | |
| --- | --- | --- | --- | --- | --- |
| **VIF for predictor variables:** | **full model** | **reduced model** |  | **full model** | **reduced model** |
| provisioning | 2.98 | 2.81 |  | 1.71 | 1.57 |
| protecting | 2.30 | 2.28 |  | 2.03 | 1.99 |
| carrying | 1.98 | 1.96 |  | 1.68 | 1.67 |
| comm. nesting, babysit, retrieval | 2.63 | 2.59 |  | 2.59 | 2.59 |
| allonursing | 1.97 | 1.96 |  | 1.60 | 1.59 |
| log mean body mass | 6.99 | 2.98 |  | 6.85 | 2.80 |
| provenance | 1.96 | 1.86 |  | 1.92 | 1.79 |
| substrate use | 2.93 | 2.68 |  | 2.79 | 2.47 |
| number of months sampled | 1.64 | 1.49 |  | 1.60 | 1.45 |
| incl. of reproductive females | 2.91 | 2.87 |  | 2.70 | 2.64 |
| log litter size | 5.96 | 3.08 |  | 5.32 | 2.66 |
| log weaning age | 2.57 | 2.08 |  | 2.71 | 2.24 |
| log gestation length | 10.82 | na |  | 10.71 | na |
| log neonatal mass | 10.51 | na |  | 10.61 | na |

**Table S2.** Variance inflation factors (VIF) of the predictor variables for female CV body mass in the full models and the reduced models, when paternal care and care provided by other group members are entered as predictor variables. Values of VIF greater than 5 indicate a problematic amount of covariance among predictors [[1](#_ENREF_1)]. For the reduced models “na” indicate that a given predictor variable has been excluded from the models due to its high VIF value in the full model.

|  | **continuous classification of paternal care and care by others** | | **binary classification of paternal care and care by others** | |
| --- | --- | --- | --- | --- |
| **VIF for predictor variables:** | **full model** | **reduced model** | **full model** | **reduced model** |
| care by others | 3.59 | 3.21 | 1.93 | 1.93 |
| paternal care | 3.29 | 3.12 | 1.98 | 1.92 |
| log mean body mass | 6.50 | 2.47 | 6.50 | 2.43 |
| provenance | 1.86 | 1.74 | 1.86 | 2.32 |
| substrate use | 2.70 | 2.42 | 2.62 | 2.32 |
| number of months sampled | 1.59 | 1.42 | 1.59 | 1.44 |
| incl. of reproductive females | 2.62 | 2.57 | 2.55 | 2.48 |
| log litter size | 5.46 | 2.63 | 4.96 | 2.57 |
| log weaning age | 2.49 | 1.96 | 2.47 | 1.98 |
| log gestation length | 10.85 | na | 9.97 | na |
| log neonatal mass | 10.43 | na | 10.29 | na |

***Estimated phylogenetic signal (λ) in the individual variables***

**Table S3.** The estimated phylogenetic signal (λ) in the individual variables using the “est.lambda()” function in the “caper” package [[2](#_ENREF_2)] in R was high for the individual allomaternal care variables and very low for CV body mass.

|  | **λ for continuous predictor variables** | **λ for binary predictor variables** |
| --- | --- | --- |
| CV body mass | <0.001 | - |
| provisioning | 0.938 | 1.000 |
| protecting | 0.913 | 0.774 |
| carrying | 1.000 | 0.848 |
| communal nesting, babysit, retrieval | 0.640 | 0.643 |
| allonursing | 0.444 | 0.507 |
| paternal care | 0.948 | 0.716 |
| care by others | 0.889 | 0.779 |

***Model sets obtained after model selection based on ∆AICc < 2***

**Table S4.** Continuous classification of allomaternal care behaviours: Model set obtained after model selection based on ∆AICc < 2 for all reduced models where gestation length and neonatal mass have been excluded to reduce multicollinearity. Best-supported models and multiple-model parameter estimates for the relationship between female CV body mass and allomaternal care behaviours. For the averaged parameter estimates and their relative explanatory importance see Table 1 in the main text.

| **explanatory variables** | | | | | | | | | | | | | **model information** | | | | | |
| --- | --- | --- | --- | --- | --- | --- | --- | --- | --- | --- | --- | --- | --- | --- | --- | --- | --- | --- |
| (Intercept) | provisioning | protecting | carrying | communal nesting | allonursing | log mean body mass | provenance | substrate use | number of months sampled | incl. of reproductive females | log litter size | log weaning age | lambda | df | log likelihood | AICc | Δ AICc | weight |
| 0.08 | -0.04 | - | - | - | - | - | 0.04 | -0.05 | - | - | 0.05 | - | 0 | 5 | 130.63 | -248.21 | 0.00 | 0.15 |
| 0.16 | -0.03 | - | - | - | - | -0.01 | 0.02 | -0.05 | - | -0.03 | - | - | 0 | 6 | 131.52 | -247.63 | 0.58 | 0.11 |
| 0.18 | -0.03 | - | - | - | - | -0.02 | - | -0.05 | - | -0.05 | - | - | 0 | 5 | 130.24 | -247.43 | 0.78 | 0.10 |
| 0.21 | -0.04 | - | - | - | - | -0.01 | - | -0.04 | -0.01 | -0.05 | - | - | 0 | 6 | 131.40 | -247.37 | 0.83 | 0.10 |
| 0.09 | -0.04 | - | - | - | - | - | 0.03 | -0.04 | - | -0.02 | 0.05 | - | 0 | 6 | 131.39 | -247.36 | 0.85 | 0.10 |
| 0.10 | -0.04 | - | - | - | - | - | 0.03 | -0.04 | -0.01 | - | 0.05 | - | 0 | 6 | 131.13 | -246.83 | 1.37 | 0.08 |
| 0.18 | -0.04 | - | - | - | - | -0.01 | 0.02 | -0.04 | -0.01 | -0.03 | - | - | 0 | 7 | 132.23 | -246.61 | 1.60 | 0.07 |
| 0.08 | -0.05 | - | 0.04 | - | - | - | 0.04 | -0.05 | - | - | 0.05 | - | 0 | 6 | 131.00 | -246.58 | 1.63 | 0.07 |
| 0.14 | -0.03 | - | - | - | - | -0.01 | 0.04 | -0.06 | - | - | - | - | 0 | 5 | 129.78 | -246.52 | 1.69 | 0.06 |
| 0.09 | -0.04 | -0.01 | - | - | - | - | 0.04 | -0.05 | - | - | 0.05 | - | 0 | 6 | 130.87 | -246.32 | 1.89 | 0.06 |
| 0.08 | -0.05 | - | - | - | 0.10 | - | 0.04 | -0.05 | - | - | 0.05 | - | 0 | 6 | 130.84 | -246.27 | 1.94 | 0.06 |
| 0.09 | -0.05 | - | - | 0.02 | - | - | 0.03 | -0.03 | - | -0.03 | 0.05 | - | 0 | 7 | 132.03 | -246.22 | 1.99 | 0.06 |

- for absence of the predictor in the model; “df”, degree of freedom; “log Likelihood”; log likelihood of the model; “AIC_c_”, Akaike’s information criterion corrected for sample size; “ΔAIC_c_”, difference in AIC_c_ between the focal model and the model with the lowest AIC_c_; “weight”, relative probability of a model within the full set of models.

**Table S5.** Continuous classification of paternal care and care provided by other group members: Model set obtained after model selection based on ∆AICc < 2 for all reduced models where gestation length and neonatal mass have been excluded to reduce multicollinearity. Best-supported models and multiple-model parameter estimates for the relationship between female CV body mass and paternal care and care by others. For the averaged parameter estimates and their relative explanatory importance see Table 2 in the main text.

| **explanatory variables** | | | | | | | | | | **model information** | | | | | | |
| --- | --- | --- | --- | --- | --- | --- | --- | --- | --- | --- | --- | --- | --- | --- | --- | --- |
| (Intercept) | care by others | paternal care | log mean body mass | provenance | substrate use | number of months sampled | incl. of reproductive females | log litter size | log weaning age | lambda | df | log likelihood | AICc | Δ AICc | weight |  |
| 0.14 | - | -0.03 | -0.01 | 0.03 | -0.06 | - | - | - | - | 0 | 5 | 130.06 | -247.06 | 0.00 | 0.15 |  |
| 0.10 | - | -0.03 | - | 0.03 | -0.04 | - | - | 0.04 | - | 0 | 5 | 129.79 | -246.52 | 0.54 | 0.11 |  |
| 0.16 | - | -0.03 | -0.01 | 0.02 | -0.05 | - | -0.02 | - | - | 0 | 6 | 131.90 | -246.39 | 0.67 | 0.11 |  |
| 0.21 | - | -0.03 | -0.01 | - | -0.04 | -0.01 | -0.03 | - | - | 0 | 6 | 130.86 | -246.29 | 0.77 | 0.10 |  |
| 0.19 | - | -0.03 | -0.01 | - | -0.05 | - | -0.04 | - | - | 0 | 5 | 129.60 | -246.15 | 0.91 | 0.10 |  |
| 0.11 | - | -0.03 | - | 0.04 | -0.05 | - | - | - | - | 0 | 4 | 128.40 | -246.06 | 1.01 | 0.09 |  |
| 0.16 | - | -0.03 | -0.01 | 0.03 | -0.05 | -0.01 | - | - | - | 0 | 6 | 130.73 | -246.03 | 1.03 | 0.09 |  |
| 0.18 | - | -0.03 | -0.01 | 0.02 | -0.04 | -0.01 | -0.02 | - | - | 0 | 7 | 131.69 | -245.53 | 1.53 | 0.07 |  |
| 0.12 | - | -0.03 | - | 0.03 | -0.04 | -0.01 | - | 0.03 | - | 0 | 6 | 130.43 | -245.43 | 1.63 | 0.07 |  |
| 0.13 | - | -0.03 | -0.01 | 0.03 | -0.05 | - | - | 0.02 | - | 0 | 6 | 130.31 | -245.20 | 1.86 | 0.06 |  |
| 0.13 | - | -0.03 | - | 0.03 | -0.04 | -0.01 | - | - | - | 0 | 5 | 129.06 | -245.07 | 1.99 | 0.06 |  |

- for absence of the predictor in the model; “df”, degree of freedom; “log Likelihood”; log likelihood of the model; “AIC_c_”, Akaike’s information criterion corrected for sample size; “ΔAIC_c_”, difference in AIC_c_ between the focal model and the model with the lowest AIC_c_; “weight”, relative probability of a model within the full set of models.

***Results of a binary coding scheme of allomaternal care behaviours as well as binary coded care provided by males (paternal care) or other group members (care by others)***

**Table S6.** Binary classification of allomaternal care behaviours: Averaged parameter estimates and their relative explanatory importance for female CV body mass (N = 87). Gestation length and neonatal mass are excluded to reduce multicollinearity between predictors.

| **predictors** | | **Relative importance of predictors** | **Model averaging estimates*** | **95% CI** |
| --- | --- | --- | --- | --- |
| intercept |  |  | 0.151 | **(0.130, 0.172)** |
| provisioning |  | 1.00 | -0.060 | **(-0.062, -0.058)** |
| protecting |  | na | 0 | 0 |
| carrying |  | na | 0 | 0 |
| communal nesting |  | 0.16 | 0.002 | (-0.001, 0.006) |
| allonursing |  | na | 0 | 0 |
| log mean body mass |  | 0.78 | -0.010 | **(-0.013, -0.007)** |
| provenance | captive | 0.70 | na | na |
|  | wild |  | 0.021 | **(0.013, 0.030)** |
| substrate use | terrestrial | 1.00 | na | na |
|  | arboreal |  | -0.049 | **(-0.052, -0.045)** |
| number of months |  | 0.27 | -0.001 | **(-0.001, -0.001)** |
| incl. of reproductive females |  | 0.68 | -0.024 | **(-0.035, -0.014)** |
| log litter size |  | 0.15 | 0.005 | (-0.002, 0.013) |
| log weaning age |  | 0.06 | 0.001 | (-0.002, 0.004) |

*: averaged model estimates based on 13 models with ΔAICc (AICc _focal model_ – AICc _best model_) < 2 since the best AICc model is not strongly weighted (weight = 0.13) [[3](#_ENREF_3)]. A full list of models is given in Table S8. Reference levels of categorical variables have an estimate of 0; na – not applicable; 95% CI - 95% confidence interval, the 95% confidence intervals that do not overlap zero are presented in bold.

**Table S7.** Binary classification of paternal care and care provided by other group members: Averaged parameter estimates and their relative explanatory importance for female CV body mass (N = 87). Gestation length and neonatal mass are excluded to reduce multicollinearity between predictors.

| **predictors** | | **Relative importance of predictors** | **Model averaging estimates*** | **95% CI** |
| --- | --- | --- | --- | --- |
| intercept |  |  | 0.155 | **(0.138, 0.173)** |
| care by others |  | 0.35 | -0.014 | **(-0.026, -0.002)** |
| paternal care |  | 0.76 | -0.035 | **(-0.046, -0.023)** |
| log mean body mass |  | 0.91 | -0.012 | **(-0.014, -0.010)** |
| provenance | captive | 0.80 | na | na |
|  | wild |  | 0.025 | **(0.018, 0.032)** |
| substrate use | terrestrial | 1.00 | na | na |
|  | arboreal |  | -0.055 | **(-0.058, -0.051)** |
| number of months |  | 0.27 | -0.001 | (-0.001, 0.001) |
| incl. of reproductive females |  | 0.48 | -0.015 | **(-0.024, -0.006)** |
| log litter size |  | 0.15 | 0.005 | (-0.001, 0.012) |
| log weaning age |  | na | 0 | 0 |

*: averaged model estimates based on 14 models with ΔAICc (AICc _focal model_ – AICc _best model_) < 2 since the best AICc model is not strongly weighted (weight = 0.13) [[3](#_ENREF_3)]. A full list of models is given in Table S9. Reference levels of categorical variables have an estimate of 0; na – not applicable; 95% CI - 95% confidence interval, the 95% confidence intervals that do not overlap zero are presented in bold.

**
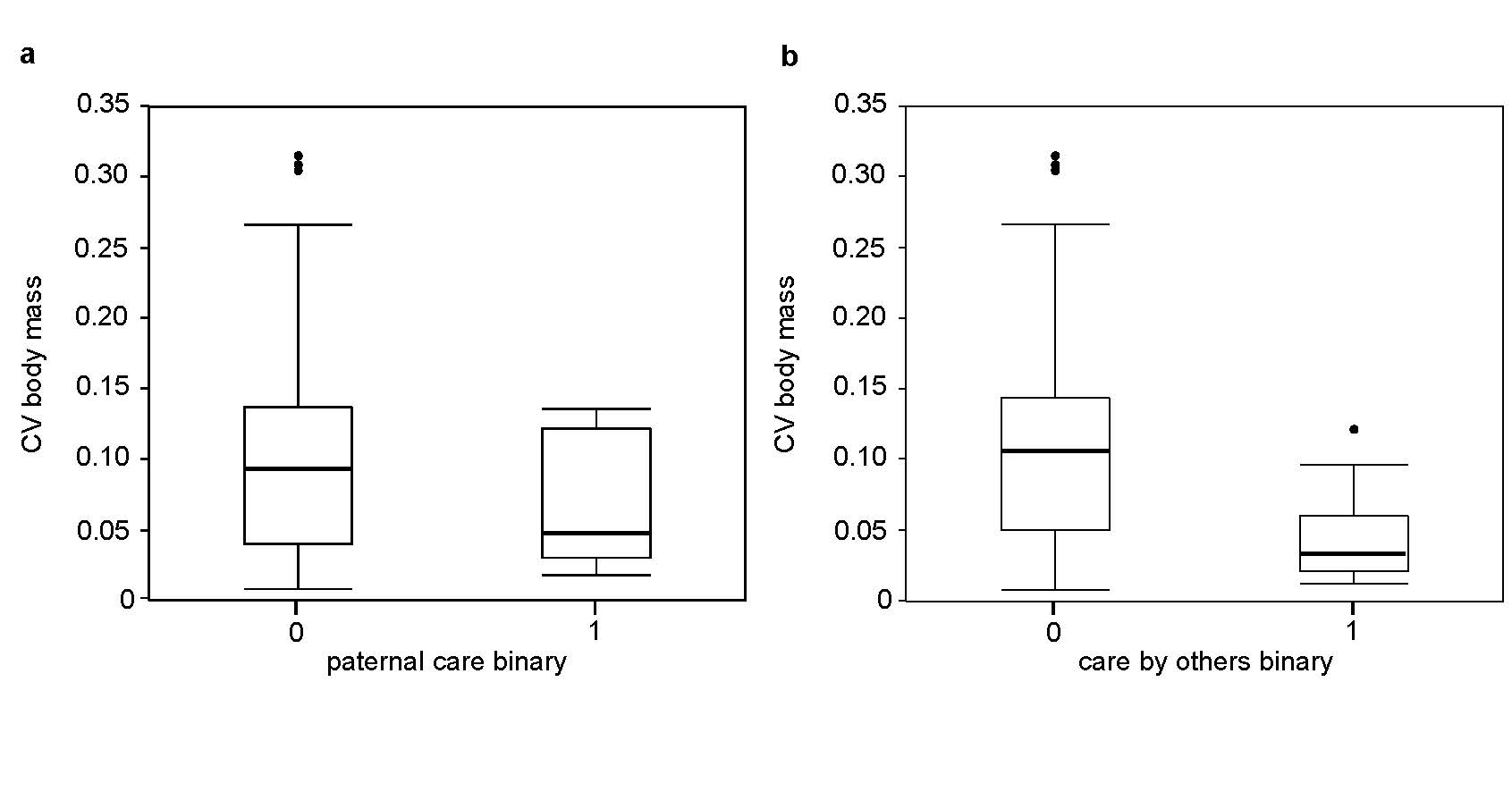
**

**Fig. S2** Female CV body mass is lower in species with paternal care (**a**) and with care provided by other group members (**b**) with the binary coding scheme. Details of phylogenetic models are shown in Table S7. Species values are listed in the Additional file 1.

**Table S8.** Binary classification of allomaternal care behaviours: Model set obtained after model selection based on ∆AICc < 2 for all reduced models where gestation length and neonatal mass have been excluded to reduce multicollinearity. Best-supported models and multiple-model parameter estimates for the relationship between female CV body mass and allomaternal care behaviours. For the averaged parameter estimates and their relative explanatory importance see Table S6.

| **explanatory variables** | | | | | | | | | | | | | **model information** | | | | | |
| --- | --- | --- | --- | --- | --- | --- | --- | --- | --- | --- | --- | --- | --- | --- | --- | --- | --- | --- |
| (Intercept) | provisioning | protecting | carrying | communal nesting | allonursing | log mean body mass | provenance | substrate use | number of months sampled | incl. of reproductive females | log litter size | log weaning age | lambda | df | log likelihood | AICc | Δ AICc | weight |
| 0.16 | -0.06 | - | - | - | - | -0.01 | 0.02 | -0.05 | - | -0.03 | - | - | 0 | 6 | 132.02 | -248.62 | 0.00 | 0.13 |
| 0.14 | -0.06 | - | - | - | - | -0.01 | 0.04 | -0.06 | - | - | - | - | 0 | 5 | 130.57 | -248.09 | 0.52 | 0.10 |
| 0.18 | -0.06 | - | - | - | - | -0.01 | - | -0.05 | - | -0.04 | - | - | 0 | 5 | 130.56 | -248.07 | 0.54 | 0.10 |
| 0.09 | -0.06 | - | - | - | - | - | 0.04 | -0.05 | - | - | 0.04 | - | 0 | 5 | 130.53 | -248.00 | 0.61 | 0.10 |
| 0.21 | -0.06 | - | - | - | - | -0.01 | - | -0.04 | -0.01 | -0.04 | - | - | 0 | 6 | 131.65 | -247.88 | 0.74 | 0.10 |
| 0.18 | -0.06 | - | - | - | - | -0.01 | 0.02 | -0.04 | -0.01 | -0.03 | 0.05 | - | 0 | 8 | 132.65 | -247.45 | 1.17 | 0.07 |
| 0.10 | -0.06 | - | - | - | - | - | 0.04 | -0.06 | - | - | - | - | 0 | 4 | 129.04 | -247.34 | 1.28 | 0.07 |
| 0.13 | -0.06 | - | - | - | - | -0.02 | 0.03 | -0.05 | - | -0.03 | 0.05 | 0.02 | 0 | 8 | 132.49 | -247.13 | 1.49 | 0.06 |
| 0.15 | -0.07 | - | - | 0.01 | - | -0.01 | 0.02 | -0.05 | - | -0.03 | 0.05 | - | 0 | 8 | 132.44 | -247.04 | 1.58 | 0.06 |
| 0.18 | -0.07 | - | - | 0.01 | - | -0.01 | - | -0.05 | - | -0.05 | - | - | 0 | 6 | 131.11 | -246.79 | 1.82 | 0.05 |
| 0.09 | -0.06 | - | - | - | - | - | 0.03 | -0.04 | - | -0.02 | 0.03 | - | 0 | 6 | 131.06 | -246.70 | 1.91 | 0.05 |
| 0.15 | -0.06 | - | - | - | - | -0.01 | 0.03 | -0.06 | -0.01 | - | 0.05 | - | 0 | 7 | 131.04 | -246.67 | 1.95 | 0.05 |
| 0.20 | -0.07 | - | - | -0.02 | - | -0.01 | - | -0.04 | -0.01 | -0.05 | - | - | 0 | 7 | 132.24 | -246.63 | 1.99 | 0.05 |

- for absence of the predictor in the model; “df”, degree of freedom; “log Likelihood”; log likelihood of the model; “AIC_c_”, Akaike’s information criterion corrected for sample size; “ΔAIC_c_”, difference in AIC_c_ between the focal model and the model with the lowest AIC_c_; “weight”, relative probability of a model within the full set of models.

**Table S9.** Binary classification of paternal care and care provided by other group members: Model set obtained after model selection based on ∆AICc < 2 for all reduced models where gestation length and neonatal mass have been excluded to reduce multicollinearity. Best-supported models and multiple-model parameter estimates for the relationship between female CV body mass and paternal care and care by others. For the averaged parameter estimates and their relative explanatory importance see Table S7.

| **explanatory variables** | | | | | | | | | | **model information** | | | | | | |
| --- | --- | --- | --- | --- | --- | --- | --- | --- | --- | --- | --- | --- | --- | --- | --- | --- |
| (Intercept) | care by others | paternal care | log mean body mass | provenance | substrate use | number of months sampled | incl. of reproductive females | log litter size | log weaning age | lambda | df | log likelihood | AICc | Δ AICc | weight |  |
| 0.14 | - | -0.05 | -0.01 | 0.03 | -0.06 | - | - | - | - | 0 | 5 | 128.75 | -244.44 | 0.00 | 0.13 |  |
| 0.16 | - | -0.05 | -0.01 | 0.03 | -0.05 | - | -0.03 | - | - | 0 | 6 | 129.85 | -244.27 | 0.17 | 0.12 |  |
| 0.08 | - | -0.06 | - | 0.04 | -0.05 | - | - | 0.04 | - | 0 | 5 | 128.43 | -243.81 | 0.64 | 0.09 |  |
| 0.19 | - | -0.04 | -0.02 | - | -0.05 | - | -0.04 | - | - | 0 | 5 | 128.39 | -243.73 | 0.71 | 0.09 |  |
| 0.15 | -0.05 | - | -0.01 | 0.03 | -0.06 | - | - | - | - | 0 | 5 | 128.32 | -243.58 | 0.86 | 0.08 |  |
| 0.21 | - | -0.05 | -0.02 | - | -0.05 | -0.01 | -0.04 | - | - | 0 | 6 | 129.24 | -243.06 | 1.38 | 0.06 |  |
| 0.14 | -0.02 | -0.03 | -0.01 | 0.03 | -0.06 | - | - | - | - | 0 | 6 | 129.18 | -242.95 | 1.50 | 0.06 |  |
| 0.16 | -0.05 | - | -0.01 | 0.03 | -0.05 | - | -0.02 | - | - | 0 | 6 | 129.17 | -242.92 | 1.53 | 0.06 |  |
| 0.12 | - | -0.05 | -0.01 | 0.03 | -0.06 | - | - | 0.02 | - | 0 | 6 | 129.09 | -242.76 | 1.68 | 0.05 |  |
| 0.16 | - | -0.05 | -0.01 | 0.03 | -0.06 | -0.01 | - | - | - | 0 | 6 | 129.09 | -242.76 | 1.69 | 0.05 |  |
| 0.18 | - | -0.05 | -0.01 | 0.02 | -0.04 | -0.01 | -0.03 | - | - | 0 | 7 | 130.29 | -242.74 | 1.70 | 0.05 |  |
| 0.17 | -0.05 | - | -0.01 | 0.03 | -0.06 | -0.01 | - | - | - | 0 | 6 | 128.97 | -242.52 | 1.93 | 0.05 |  |
| 0.16 | -0.02 | -0.03 | -0.01 | 0.03 | -0.05 | - | -0.03 | - | - | 0 | 7 | 130.16 | -242.48 | 1.96 | 0.05 |  |
| 0.21 | -0.05 | - | -0.02 | - | -0.05 | -0.01 | -0.04 | - | - | 0 | 6 | 128.95 | -242.47 | 1.97 | 0.05 |  |

- for absence of the predictor in the model; “df”, degree of freedom; “log Likelihood”; log likelihood of the model; “AIC_c_”, Akaike’s information criterion corrected for sample size; “ΔAIC_c_”, difference in AIC_c_ between the focal model and the model with the lowest AIC_c_; “weight”, relative probability of a model within the full set of models.

***Results for the subset of studies including only wild-caught females***

The subset of studies including only wild-caught females has been analysed using a continuous classification of care behaviours. Results using a binary coding scheme are very similar (not shown).

**Table S10.** Variance inflation factors (VIF) of the allomaternal care behaviours as predictor variables for female CV body mass in the full models and the reduced models, when all allomaternal care behaviours are entered separately as predictors, for the subset only including wild-caught females (N = 49). Values of VIF greater than 5 indicate a problematic amount of covariance among predictors [[1](#_ENREF_1)]. For the reduced models “na” indicate that a given predictor variable has been excluded from the models due to its high VIF value in the full model.

| **VIF for predictor variables:** | **full model** | **reduced model** |  |
| --- | --- | --- | --- |
| provisioning | 3.81 | 3.73 |  |
| protecting | 2.80 | 2.67 |  |
| carrying | 2.25 | 2.11 |  |
| comm. nesting, babysit, retrieval | 3.26 | 3.24 |  |
| allonursing | 1.79 | 1.77 |  |
| log mean body mass | 6.83 | 3.43 |  |
| substrate use | 1.46 | 1.33 |  |
| number of months sampled | 1.73 | 1.41 |  |
| incl. of reproductive females | 1.67 | 1.60 |  |
| log litter size | 4.36 | 2.68 |  |
| log weaning age | 2.15 | 1.72 |  |
| log gestation length | 11.59 | na |  |
| log neonatal mass | 10.45 | na |  |

**Table S11.** Variance inflation factors (VIF) of the predictor variables for female CV body mass in the full models and the reduced models, when paternal care and care provided by other group members are entered as predictor variables, for the subset only including wild-caught females (N = 49). Values of VIF greater than 5 indicate a problematic amount of covariance among predictors [[1](#_ENREF_1)]. For the reduced models “na” indicate that a given predictor variable has been excluded from the models due to its high VIF value in the full model.

|  | **continuous classification of paternal care and care by others** | |
| --- | --- | --- |
| **VIF for predictor variables:** | **full model** | **reduced model** |
| care by others | 3.55 | 3.22 |
| paternal care | 3.39 | 3.13 |
| log mean body mass | 5.14 | 2.27 |
| substrate use | 1.36 | 1.21 |
| number of months sampled | 1.44 | 1.18 |
| incl. of reproductive females | 1.39 | 1.30 |
| log litter size | 3.78 | 1.85 |
| log weaning age | 1.98 | 1.56 |
| log gestation length | 11.30 | na |
| log neonatal mass | 10.21 | na |

**Table S12.** Allomaternal care behaviours: Averaged parameter estimates and their relative explanatory importance for female CV body mass in a subset only including wild-caught females (N = 49).

| **predictors** | | **Relative importance of predictors** | **Model averaging estimates*** | **95% CI** |
| --- | --- | --- | --- | --- |
| intercept |  |  | 0.140 | **(0.124, 0.156)** |
| provisioning |  | 0.85 | -0.038 | **(-0.048, -0.027)** |
| protecting |  | 0.27 | -0.011 | (-0.022, 0.001) |
| carrying |  | na | 0 | 0 |
| communal nesting |  | na | 0 | 0 |
| allonursing |  | na | 0 | 0 |
| log mean body mass |  | 0.21 | -0.002 | (-0.005, 0.001) |
| substrate use | terrestrial | 0.85 | na | na |
|  | arboreal |  | -0.045 | **(-0.057, -0.033)** |
| number of months sampled |  | na | 0 | 0 |
| incl. of reproductive females |  | 0.26 | -0.009 | (-0.019, 0.001) |
| log litter size |  | 0.44 | 0.026 | **(0.008, 0.043)** |
| log weaning age |  | na | 0 | 0 |

*: averaged model estimates based on 12 models with ΔAICc (AICc _focal model_ – AICc _best model_) < 2 since the best AICc model is not strongly weighted (weight = 0.15) [[3](#_ENREF_3)]. A full list of models is given in Table S14. Reference levels of categorical variables have an estimate of 0; na – not applicable; 95% CI - 95% confidence interval, the 95% confidence intervals that do not overlap zero are presented in bold.

**Table S13.** Paternal care and care provided by other group members: Averaged parameter estimates and their relative explanatory importance for female CV body mass in a subset only including wild-caught females (N = 49).

| **predictors** | | **Relative importance of predictors** | **Model averaging estimates*** | **95% CI** |
| --- | --- | --- | --- | --- |
| intercept |  |  | 0.147 | **(0.119, 0.175)** |
| care by others |  | na | 0 | 0 |
| paternal care |  | 1.00 | -0.038 | **(-0.041, -0.036)** |
| log mean body mass |  | 0.23 | -0.002 | (-0.007, 0.003) |
| substrate use | terrestrial | 0.86 | na | na |
|  | arboreal |  | -0.047 | **(-0.074, -0.020)** |
| number of months |  | na | 0 | 0 |
| incl. of reproductive females |  | na | 0 | 0 |
| log litter size |  | 0.43 | 0.022 | (-0.008, 0.052) |
| log weaning age |  | na | 0 | 0 |

*: averaged model estimates based on 4 models with ΔAICc (AICc _focal model_ – AICc _best model_) < 2 since the best AICc model is not strongly weighted (weight = 0.34) [[3](#_ENREF_3)]. A full list of models is given in Table S15. Reference levels of categorical variables have an estimate of 0; na – not applicable; 95% CI - 95% confidence interval, the 95% confidence intervals that do not overlap zero are presented in bold.

**Table S14.** Allomaternal care behaviours: Model set obtained after model selection based on ∆AICc < 2 for all reduced models where gestation length and neonatal mass have been excluded to reduce multicollinearity. Best-supported models and multiple-model parameter estimates for the relationship between female CV body mass and allomaternal care behaviours for the subset of studies including only wild-caught females (N = 49). For the averaged parameter estimates and their relative explanatory importance see Table S12.

| **explanatory variables** | | | | | | | | | | | | | **model information** | | | | | |
| --- | --- | --- | --- | --- | --- | --- | --- | --- | --- | --- | --- | --- | --- | --- | --- | --- | --- | --- |
| (Intercept) | provisioning | protecting | carrying | communal nesting | allonursing | log mean body mass | provenance | substrate use | number of months sampled | incl. of reproductive females | log litter size | log weaning age | lambda | df | log likelihood | AICc | Δ AICc | weight |
| 0.12 | -0.05 | - | - | - | - | - | 0.03 | -0.05 | - | - | 0.05 | - | 0 | 5 | 63.54 | -115.68 | 0.00 | 0.15 |
| 0.14 | -0.04 | - | - | - | - | - | 0.03 | -0.05 | - | - | - | - | 0 | 4 | 62.26 | -115.61 | 0.07 | 0.14 |
| 0.10 | -0.05 | - | - | - | - | - | 0.04 | - | - | - | 0.06 | - | 0 | 4 | 61.87 | -114.83 | 0.85 | 0.10 |
| 0.17 | -0.04 | - | - | - | - | -0.01 | 0.04 | -0.05 | - | - | - | - | 0 | 5 | 62.97 | -114.55 | 1.13 | 0.08 |
| 0.12 | -0.05 | - | - | - | - | - | 0.04 | -0.05 | - | -0.04 | 0.06 | - | 0 | 6 | 64.27 | -114.53 | 1.15 | 0.08 |
| 0.15 | - | -0.05 | - | - | - | - | - | -0.06 | - | - | - | - | 0 | 3 | 61.71 | -114.51 | 1.17 | 0.08 |
| 0.19 | - | -0.05 | - | - | - | -0.01 | - | -0.06 | - | - | - | - | 0 | 4 | 62.73 | -114.06 | 1.62 | 0.07 |
| 0.15 | -0.04 | - | - | - | - | - | 0.02 | -0.05 | - | -0.03 | - | - | 0 | 5 | 62.66 | -113.93 | 1.75 | 0.06 |
| 0.15 | -0.03 | -0.03 | - | - | - | - | 0.04 | -0.06 | - | - | - | - | 0 | 5 | 62.64 | -113.89 | 1.79 | 0.06 |
| 0.19 | -0.04 | - | - | - | - | -0.01 | 0.04 | -0.05 | - | -0.04 | - | - | 0 | 6 | 63.92 | -113.85 | 1.84 | 0.06 |
| 0.11 | -0.05 | - | - | - | - | - | 0.02 | - | - | -0.04 | 0.07 | - | 0 | 4 | 62.58 | -113.77 | 1.91 | 0.06 |
| 0.12 | -0.04 | -0.02 | - | - | - | - | 0.03 | -0.05 | - | - | 0.05 | - | 0 | 6 | 63.87 | -113.74 | 1.94 | 0.06 |

- for absence of the predictor in the model; “df”, degree of freedom; “log Likelihood”; log likelihood of the model; “AIC_c_”, Akaike’s information criterion corrected for sample size; “ΔAIC_c_”, difference in AIC_c_ between the focal model and the model with the lowest AIC_c_; “weight”, relative probability of a model within the full set of models.

**Table S15.** Paternal care and care provided by other group members: Model set obtained after model selection based on ∆AICc < 2 for all reduced models where gestation length and neonatal mass have been excluded to reduce multicollinearity. Best-supported models and multiple-model parameter estimates for the relationship between female CV body mass and paternal care and care by others for the subset of studies including only wild-caught females (N = 49). For the averaged parameter estimates and their relative explanatory importance see Table S13.

| **explanatory variables** | | | | | | | | | | **model information** | | | | | | |
| --- | --- | --- | --- | --- | --- | --- | --- | --- | --- | --- | --- | --- | --- | --- | --- | --- |
| (Intercept) | care by others | paternal care | log mean body mass | provenance | substrate use | number of months sampled | incl. of reproductive females | log litter size | log weaning age | lambda | df | log likelihood | AICc | Δ AICc | weight |  |
| 0.15 | - | -0.04 | - | 0.03 | -0.06 | - | - | - | - | 0 | 4 | 62.76 | -116.60 | 0.00 | 0.34 |  |
| 0.13 | - | -0.04 | - | 0.02 | -0.05 | - | - | 0.05 | - | 0 | 5 | 63.83 | -116.26 | 0.35 | 0.29 |  |
| 0.18 | - | -0.04 | -0.01 | - | -0.06 | - | - | - | - | 0 | 4 | 63.59 | -115.78 | 0.83 | 0.23 |  |
| 0.11 | - | -0.04 | - | 0.03 | - | - | - | 0.06 | - | 0 | 4 | 61.85 | -114.79 | 1.81 | 0.14 |  |

- for absence of the predictor in the model; “df”, degree of freedom; “log Likelihood”; log likelihood of the model; “AIC_c_”, Akaike’s information criterion corrected for sample size; “ΔAIC_c_”, difference in AIC_c_ between the focal model and the model with the lowest AIC_c_; “weight”, relative probability of a model within the full set of models.

***References***

1. Rogerson P. Statistical Methods for Geography. London: Sage; 2001.

2. Orme D. The caper package: comparative analysis of phylogenetics and evolution in R. R package version 2013;5.

3. Symonds MR, Moussalli A. A brief guide to model selection, multimodel inference and model averaging in behavioural ecology using Akaike’s information criterion. Behav Ecol Sociobiol 2011;65:13-21.
