# Supplementary figures and images for: Getting fat or getting help? How female mammals cope with energetic constraints on reproduction
Source: Front Zool. 2017 Jun 12;14:29. doi: 10.1186/s12983-017-0214-0 (PMC5468974; doi:10.1186/s12983-017-0214-0)

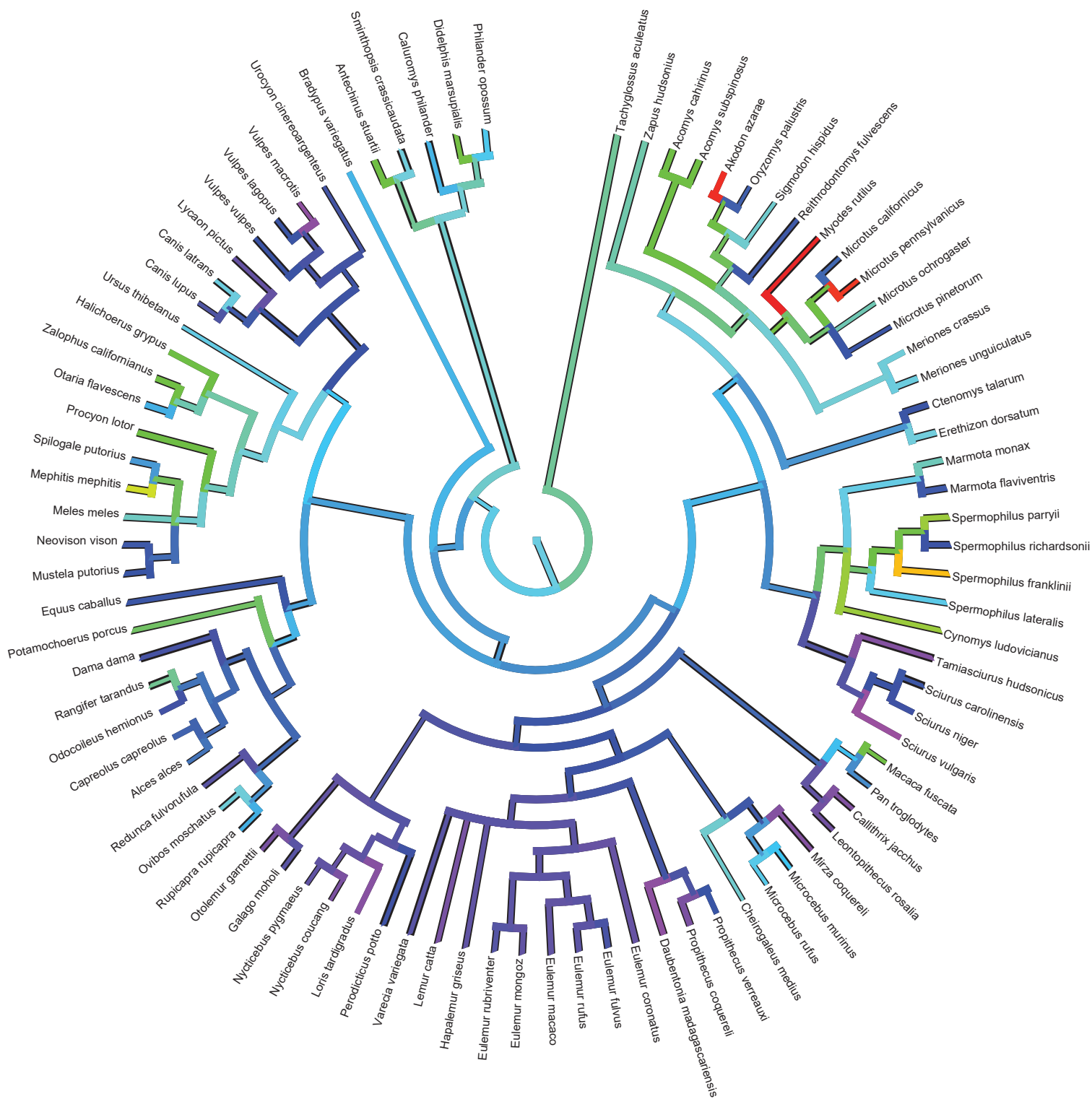

CV body mass

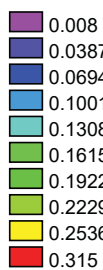

Supplement: Supplementary file 5 — Phylogenetic tree of 87 mammal species used in this study visualised using Mesquite v. 3.11 [126]. (PDF 83 kb) [file 12983_2017_214_MOESM5_ESM.pdf]
